# The Role of Multifocal Intraocular Lens Implantation in Extreme Axial Myopia: A Case Report and Literature Review

**DOI:** 10.7759/cureus.33976

**Published:** 2023-01-19

**Authors:** Stephen A LoBue, Thomas M Catapano, Brittany B DeNaro, Christopher Shelby, Wyche T Coleman

**Affiliations:** 1 Department of Ophthalmology, Willis-Knighton, Shreveport, USA; 2 Department of Ophthalmology, St. George’s University, School of Medicine, West Indies, GRD; 3 Department of Ophthalmology, Louisiana State University Health Sciences Center, Shreveport, USA

**Keywords:** high myopia, lasik, multifocal intraocular lenses, cataract extraction, extreme myopia

## Abstract

The prevalence of high myopia is rising globally. In addition to an increased risk of retinal detachment, high myopia is associated with earlier cataract formation. Patients with myopia are also often more motivated to become spectacle-independent after a lens procedure. However, the use of multifocal intraocular lens (MfIOL) remains controversial for patients with extreme myopia, which is classified as patients with an axial length >28 mm. Here, we present the case of a 64-year-old patient with visually significant cataract and extreme axial myopia >31 mm in both eyes who desired to be spectacle-independent. A preoperative workup revealed a normal macula with peripheral lattice degeneration. On optical coherence tomography, the macula had a normal fovea contour without the presence of a staphyloma. A thorough peripheral examination was performed by a retina specialist which required no prophylactic treatment. Pentacam analysis demonstrated a low spherical aberration and minimal ectasia risk. Cataract surgery was uneventful with a 5 mm laser capsulotomy centered over the visual axis with the placement of a trifocal intraocular lens. Two months after the cataract surgery, the remaining refractive error was corrected with a laser-assisted in situ keratomileusis enhancement. The patient achieved an uncorrected distance visual acuity of 20/15- and uncorrected near visual acuity of J1+ in both eyes. Overall, this case report and review aims to highlight important preoperative, intraoperative, and postoperative techniques to improve patient outcomes with MfIOL in patients with extreme myopia.

## Introduction

The second most common cause of blindness and distance visual impairment globally is a refractive error from myopia [1]. The estimated prevalence of myopia in 2000 was 1.4 billion people or 22.9% of the global population [2]. It is predicted that the prevalence of myopia will increase to 4.76 billion people or 50% of the global population by 2050 [2]. Approximately 938 million people will be classified as having high myopia with a refractive error of at least -5 diopters [2].

However, high myopia is associated with a greater risk of irreversible vision loss from glaucoma, retinal detachment, myopic macular degeneration, and cataracts [3]. Regarding cataract formation, the Blue Mountains Eye Study found that high myopia was associated with an increased incidence of nuclear cataract formation (adjusted odds ratio (OR) = 3.01, 95% confidence interval (CI) = 1.35-6.71) and posterior subcapsular cataract (OR = 7.80, CI = 3.51-17.35) [4]. Between all spectrums of myopia, high myopia was associated with the highest incidence of cataract surgery (OR = 4.81, CI = 2.33-9.93) [4].

Nevertheless, cataract surgery can be more challenging in high myopes due to the increased or fluctuating depth of the anterior chamber, large or floppy capsular bag, potential zonular weakness, and higher rates of retinal detachment [5].

However, patients with myopia are often more motivated to become spectacle or contact lens independent after a lens procedure. Yet, the use of multifocal intraocular lens (MfIOL) remains controversial for patients with extreme myopia, which is classified as an axial length >28 mm. Retinal pathology or lens decentration may limit optimal visual potential, inhibiting the performance of a MfIOL. Few studies have analyzed the performance of MfIOL in patients with extreme myopia. Therefore, we present a case report and review regarding extreme myopia and MfIOL performance.

## Case presentation

A 64-year-old male presented to the Willis-Knighton Eye Institute for evaluation for cataract surgery. The patient complained of worsening glare and decreased nighttime vision. He had an ocular history of high myopia and lattice degeneration. A best-corrected distance visual acuity (BCDVA) was 20/25-2 for the right eye (OD) and 20/40+2 for the left eye (OS), with a manifest refraction of -16.75 + 0.25 × 145 OD and -15.50 + 0.75 × 180 OS.

The slit lamp examination was unremarkable besides 2+ nuclear sclerosis in both eyes (OU). The intraocular pressure (IOP) was 16 mmHg bilaterally. The dilated fundus examination noted a 0.4 cup-to-disc ratio with 360 peripapillary atrophy, normal macula, and lattice degeneration in the far periphery OU.

**Figure 1:**
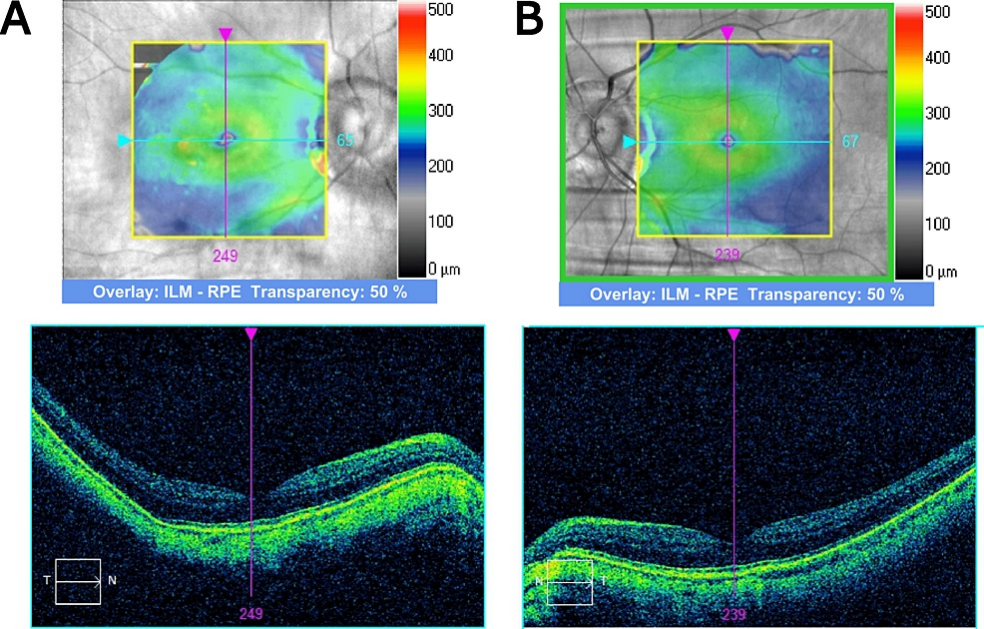
Macula optical coherence tomography. Macula optical coherence tomography of the (A) right eye and (B) left eye. A normal foveal contour was documented without degeneration or edema in the neurosensory retina in both eyes.

Cirrus spectral-domain macular optical coherence tomography (OCT) (Carl Zeiss Meditec AG, Germany) demonstrated normal foveal contour, neurosensory retina, and retinal pigment epithelium without any signs of retinal degeneration or edema in both eyes (Figure 1).

**Figure 2:**
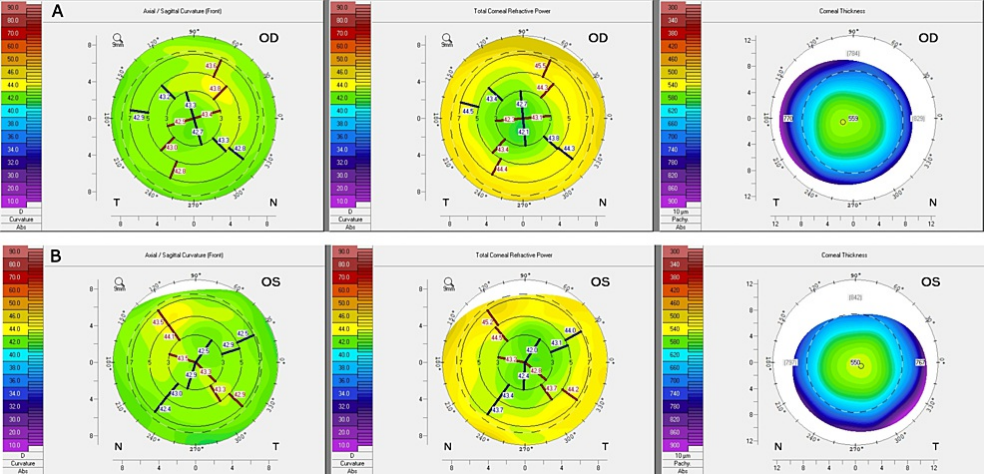
Corneal topography. Pentacam corneal topography demonstrates minimal corneal astigmatism in the (A) right eye and (B) left eye with the thinnest central pachymetry of 559 μm OD and 550 μm OS. OD = right eye; OS = left eye

Pentacam (Oculus, Wetzlar, Germany) analysis demonstrated minimal corneal astigmatism OU with the thinnest central pachymetry of 559 μm OD and 550 μm OS (Figure 2).

Examining the right eye, the total corneal refractive power at 4 mm was 0.2 D at 83.4 versus 0.3 at 13 with the wavefront aberration (WFA) 4 mm zone. Total cornea spherical aberration in the 6 mm zone was 0.377. Examining the left eye, the total corneal refractive power at 4 mm was 0.5 D at 137.5 versus 0.2 at 153 with the WFA 4 mm zone. Total cornea spherical aberration in the 6 mm zone was 0.324.

**Figure 3:**
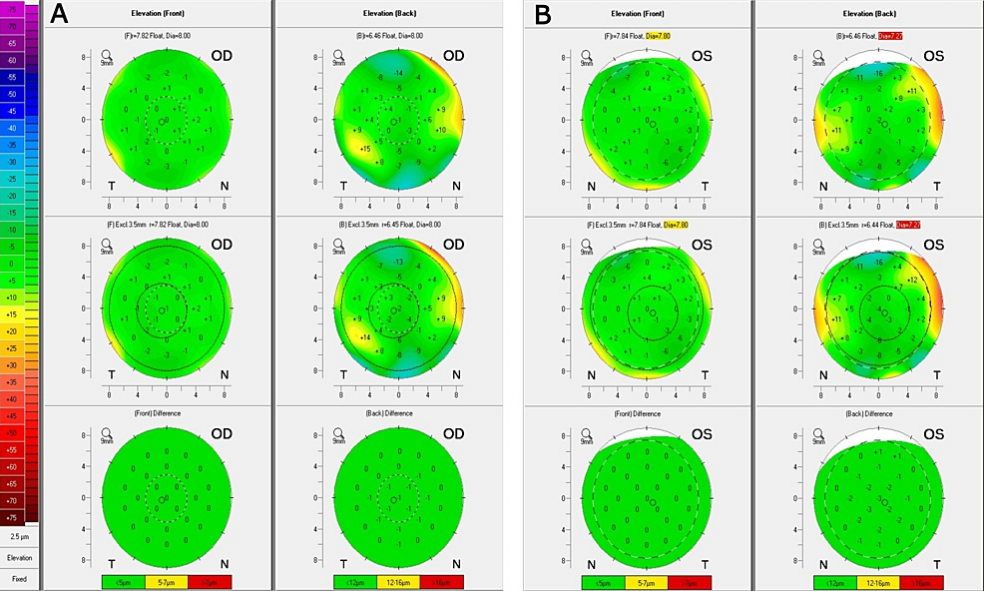
Corneal tomography. Belin/Ambrósio enhanced ectasia display demonstrates normal levels of anterior and posterior corneal elevation in the (A) right eye and (B) left eye. No signs of underlying ectasia risks are observed in either eye.

The Belin/Ambrósio enhanced ectasia display demonstrated normal levels of anterior and posterior corneal elevation OU. No signs of underlying ectasia risks were observed in either eye (Figure 3).

IOL master 700 (Carl Zeiss Meditec AG, Germany) revealed an axial length of 31.01 mm OD and 31.06 mm OS.

The patient desired spectacle independence and was a candidate for a trifocal intraocular lens (Panoptix, Alcon, Fort Worth, TX). Aiming for Plano OU, the patient required a two-diopter IOL OD and a three-diopter IOL OS. Because the trifocal ranged from +6 to +30D, a +6D lens was selected for both eyes with the plan for laser-assisted in situ keratomileusis (LASIK) enhancement OU.

Femtosecond laser-assisted lens surgery using the LenSx platform (Alcon, Fort Worth, TX) was used to create a 5 mm anterior capsulotomy and fragmentation of the nucleus. Phacoemulsification was performed using the Centurion Vision System (Alcon, Fort Worth, TX) under topical anesthesia. Optiwave Refractive Analysis (ORA) (Alcon Fort Worth, TX) was used after cataract extraction to verify preoperative IOL selection.

Cataract surgery was uncomplicated and completed on 8/18/20 OD and 8/25/20 OS. Approximately one month after cataract surgery, BCDVA was 20/25+2 OD and 20/20-2 OS with a manifest refraction of -2.75 OD and -1.25 OS. The patient received YAG capsulotomy OU for 1+ posterior capsular opacification.

At two month follow-up, uncorrected distance visual acuity (UDVA) was measured at 20/100 OD and 20/40 OS with uncorrected near visual acuity (UNVA) at J1+ OU. BCDVA was 20/20 OU with manifest refraction -3.00 + 0.50 × 170 OD and -1.75 + 0.75 × 170 OS. LASIK was performed bilaterally aiming for Plano.

Postoperative day one post-LASIK, the patient had a UDVA of 20/20-1 OU and UNVA of J2+ OU. One month post-LASIK, UDVA was 20/15- OU and UNVA was J1+ OU. The patient was seen one year later with a stable UDVA of 20/15- OU and UNVA of J1+ OU. The patient was content with his visual acuity and achieved spectacle independence. The patient is now being followed annually.

## Discussion

The prevalence of myopia and high myopia will significantly increase globally to approximately five billion and one billion people, respectively, by 2050 [2]. High myopia is also associated with earlier cataract formation, which may pose an increased risk of complications during cataract extraction. Furthermore, as cataract surgery transitions to a refractive procedure with a higher percentage of patients requesting spectacle independence, more pressure will be placed on the surgeon to achieve a perfect result. Hence, this case report and review will highlight important preoperative, intraoperative, and postoperative techniques to improve patient outcomes with MfIOLs in patients with extreme myopia.

Various IOL calculations can be used for high and extreme myopia. One retrospective case series examined 73 eyes with an average axial length ± standard deviation of 31.17 ± 1.43 mm [6]. Prediction errors were calculated and compared between different formulas to evaluate the accuracy. The accuracy of the Emmetropia Verifying Optical (EVO) version 2.0, Kane, Barrett Universal II, and Olsen formulas was comparable and significantly better than that of the SRK/T and Haigis formulas in reducing refractive mean error and absolute mean error [6]. Similarly, a study examining 410 eyes found no significant difference in the deviation in predictive refractive error between EVO 2.0, Kane, Barrett Universal II, and Olsen among all axial length subgroups [7]. Once again, Olsen (47.1%), Barrett (45.9%), Kane (45.4%), and EVO 2.0 (45.1%) formulas had greater proportions of predictive refractive error within ±0.25 D compared to Hoffer Q (35.9%), SRK/T (35.9%), and Holladay 1 (33.4%) formulas (p < 0.05) [7].

A similar trend was also noted in high myopes with trifocal IOL calculations. The Barrett Universal II, Olsen, Kane, and EVO 2.0 formulas produced a statistically lower median absolute error than the SRK/T formulas (p < 0.05) [8]. Moreover, the Barrett Universal II formula produced higher percentages of eyes within ±0.50 D of the prediction error compared to the SRK/T formula (p < 0.05). However, there were no significant differences in the prediction accuracy of all formulas tested in axial lengths less than 28 mm [8].

Additional IOL calculations can be determined during surgery using intraoperative aberrometry (ORA). Soifer et al. documented that in highly myopic eyes with an axial length of greater than 27 mm, ORA demonstrated a trend toward lower mean absolute error when compared to the Barrett Universal II formula (p = 0.076) [9].

Other considerations during preoperative testing should include a thorough retina examination. In addition to a retinal referral to evaluate for peripheral pathology, a close examination of the posterior pole should be performed by the surgeon. Macular and optic nerve pathology needs to be excluded as decreased contrast sensitivity can adversely affect lens performance [10]. Macular OCT plays a crucial role in ruling out abnormalities including edema or degeneration which would negatively affect lens performance. Circumscribed outpouching of the wall of the globe, also known as a posterior staphyloma, is the most common cause of maculopathy among pathologic myopia. However, the severity of a posterior staphyloma can vary and does not completely exclude a patient from a MfIOL but should cause hesitation in the surgeon. One study examining extreme myopes with and without staphylomas for AT LISA trifocal IOL (Carl Zeiss, Meditec) implantation found superior visual outcomes in eyes without a staphyloma [11]. Corrective distance acuity was 0.91 and 0.74 for the negative versus positive staphyloma group, respectively. Specifically, nasal-inferior macula staphyloma with higher macula plane tilt (>30 degrees) showed worse outcomes [11].

Intraoperatively, the capsulorhexis position is vital for optimal performance. Eyes with eccentric capsulorhexis may be more predisposed to decentration [12]. The size of the capsulorhexis is also crucial as patients with high myopia tend to have larger and more floppy capsular bags. Nevertheless, too small of a capsulorhexis may increase the risk of postoperative capsular contraction, causing lens malposition [12,13]. Optimal capsulorhexis is between 5 mm and 5.5 mm so that 360-degree coverage of the lens exists with the anterior capsule. To standardize this size we perform laser capsulotomy on all of our myopic patients. However, this could be standardized by a well-experienced surgeon with adequately marked utrata forceps, corneal markings, or advanced imaging overlay.

IOL stability within the capsular bag is also essential for optimal lens performance [14]. IOL decentration and tilt can lead to increased higher-order aberrations (HOAs) including coma and spherical aberrations which reduce visual quality [14]. Specifically, high coma aberrations have been associated with monocular diplopia while spherical aberration has been associated with increased glare [15,16]. MfIOL is very sensitive to lens decentration. One study examining diffractive bifocal (AT LISA 809M; Carl Zeiss Meditec) and trifocal IOL (AT LISA 839M; Carl Zeiss Meditec) found significantly reduced optical quality at all distances if decentration exceeded 0.75 mm [17].

We place extra precautions with our MfIOL centration after cataract extraction. During centration, we ask the patient to focus on the most medial Purkinje light reflex of the operating microscope so that the MfIOL is centered on the visual axis and not the pupil axis. We also slightly underfill the eye so that the intraocular pressure is around 8-10 mmHg to have more adherence with the IOL and the capsular bag.

The stability and centration of MfIOLs in high myopes may be affected by the lens design. Common IOL designs include C-loop versus plate-haptic design.

Studies have associated C-loop haptics (Tecnis ZMB00 multifocal IOL) with significantly worse vertical decentration compared to plate haptics (Zeiss AT LISA tri 839MP lens) [5,18]. Vertical decentration was associated with worse aberration data (increased HOAs, coma, trefoil) and more subjective symptoms [5,18].

It is hypothesized that decentration from C-loop designs occurs due to the friction between the two haptics and the larger capsular bag is not strong enough to compensate for the gravity of the IOL. Lack of vertical support is especially seen when the IOL is horizontally placed [18]. As a result, MfIOLs of this haptic design may settle inferiorly slightly in highly myopic eyes. However, unlike the C-loop design, the plate-haptic design omits the gap between the optic and haptics and gains greater support from the capsular bag through its four corners. This implies that the IOL is held tightly by the capsular bag, which better addresses the challenges of gravity at any of the in-bag placements.

However, C-loop design MfIOL such as diffractive trifocal toric IOL (PanOptix Toric) and diffractive bifocal toric IOL (AcrySof ReSTOR SND1T, Alcon, Fort Worth, USA) have been documented to perform well in patients with moderate to high myopia at any orientation [19]. The study included 120 eyes with astigmatism with axial lengths from 24.0 mm to 26.5 mm. Postoperative examinations measured near, intermediate, and distance visual acuity, binocular defocus curves, and patient satisfaction via a standardized questionnaire. Both lenses performed equally well with significantly better intermediate vision in the trifocal group.

Refractive misses, or in our case limitation of MfIOL power, will require surgeons to enhance their patients with photorefractive keratectomy or LASIK. However, due to the increased underlying risk in high and extreme myopia, surgeons may be hesitant to perform LASIK for fear of causing a retinal detachment after significant elevation in IOP during suction. The occurrence of rhegmatogenous retinal detachment in the myopic population is estimated at around 2.2% compared to 0.1% in the emmetropic population [20].

A large multicenter retrospective study examining 22,700 eyes from 1994 to 2002 found 15 eyes of 13 patients developed rhegmatogenous retinal detachment after refractive surgery. Eight cases had LASIK, six had photorefractive keratectomy, and one had LASIK on top of photorefractive keratectomy. The average myopia was -13.5 D with a mean occurrence at 20 months after refractive surgery [20].

The low incidence in the above study of <0.1% may be explained by patient selection, systematic monitoring via fundus examinations, and the prophylactic treatment of peripheral degenerative lesions by photocoagulation [20]. Thus, refractive surgery on severe myopia can be safely done with adequate clinical monitoring and prophylactic intervention if needed.

Our patient was an extreme myope with an axial length >31 mm who desired to be spectacle-independent. A preoperative workup revealed a normal macula with peripheral degenerative changes. On OCT, the macula had a normal fovea contour without the presence of a staphyloma. A thorough peripheral examination was performed by a retina specialist which required no prophylactic treatment. Pentacam analysis demonstrated a low spherical aberration and minimal ectasia risk. Cataract surgery was uneventful with a 5 mm laser capsulotomy centered over the visual axis. Intraoperative IOL calculations were confirmed with ORA and the trifocal IOL was centered over the visual axis. Two months post-cataract surgery, the remaining refractive error was corrected with a LASIK enhancement. The patient was content with his visual acuity and achieved spectacle independence.

## Conclusions

The prevalence of high myopia is rising globally. In addition to an increased risk of retinal detachment, high myopia is associated with earlier cataract formation. Furthermore, as a higher percentage of myopic patients request spectacle independence, more pressure will be placed on the surgeon to achieve a perfect result.

Although limited, the literature may support adequate results of MfIOL placement in select patients with high or extreme levels of myopia. Good outcomes mirror thorough preoperative planning, testing, and laser prophylaxis when applicable. It is also imperative patients are a candidate for laser refractive enhancement as extreme myopia patients are at a higher risk for refractive errors. Extreme myopia should not be a contraindication for MfIOL selection but rather a checkpoint for closer examination and evaluation. Nevertheless, larger studies are needed to compare the performance of MfIOL in extreme myopes to further optimize visual outcomes.
